# The Nutritional Phenotyping of Idiopathic Pulmonary Fibrosis Through Morphofunctional Assessment: A Bicentric Cross-Sectional Case–Control Study

**DOI:** 10.3390/life15040516

**Published:** 2025-03-21

**Authors:** Alicia Sanmartín-Sánchez, Rocío Fernández-Jiménez, Eva Cabrera-César, Francisco Espíldora-Hernández, Isabel Vegas-Aguilar, María del Mar Amaya-Campos, Fiorella Ximena Palmas-Candia, Josefina Olivares-Alcolea, Víctor José Simón-Frapolli, Isabel Cornejo-Pareja, Ana Sánchez-García, Mora Murri, Patricia Guirado-Peláez, Álvaro Vidal-Suárez, Lourdes Garrido-Sánchez, Francisco J. Tinahones, Jose Luis Velasco-Garrido, Jose Manuel García-Almeida

**Affiliations:** 1 Department of Endocrinology and Nutrition, Son Espases University Hospital, 07120 Mallorca, Spain; alicia.sanmartin@outlook.es (A.S.-S.); jolivares@ssib.es (J.O.-A.); 2 Department of Endocrinology and Nutrition, Virgen de la Victoria University Hospital, 29010 Malaga, Spain; isabel.mva13@gmail.com (I.V.-A.); mariadelmarac2@gmail.com (M.d.M.A.-C.); isabelmaria_cornejo@hotmail.com (I.C.-P.); anaasanchez12@gmail.com (A.S.-G.); moramurri@gmail.com (M.M.); pguirado1991@gmail.com (P.G.-P.); alvarovidal1992@gmail.com (Á.V.-S.); lourdes.garrido@ibima.eu (L.G.-S.); fjtinahones@uma.es (F.J.T.); jgarciaalmeida@gmail.com (J.M.G.-A.); 3 Instituto de Investigación Biomédica de Málaga y Plataforma en Nanomedicina-IBIMA Plataforma BIONAND, 29010 Malaga, Spain; 4 Department of Medicine and Dermatology, Málaga University, 29016 Malaga, Spain; 5 Department of Endocrinology and Nutrition, Quironsalud Málaga Hospital, Av. Imperio Argentina, 29004 Malaga, Spain; 6 Department of Neumology, Virgen de la Victoria University Hospital, 29010 Malaga, Spain; evacabreracesar@gmail.com (E.C.-C.); jlvelascogarrido@hotmail.com (J.L.V.-G.); 7 Department of Neumology, Regional University Hospital, 29010 Malaga, Spain; fespildorahernandez@gmail.com; 8 Endocrinology and Nutrition Department, Vall D’Hebron University Hospital, 08035 Barcelona, Spain; fiorellaximena.palmas@vallhebron.cat; 9 Centro de Investigación Biomédica en Red Fisiopatología de la Obesidad y Nutrición (CIBEROBN), Carlos III Health Institute (ISCIII), Málaga University, 29010 Malaga, Spain; 10 Instituto de Investigación Biomédica de Málaga y Plataforma en Nanomedicina-IBIMA Plataforma BIONAND, Heart Area, Virgen de la Victoria University Hospital, 29010 Malaga, Spain

**Keywords:** idiopathic pulmonary fibrosis, morphofunctional assessment, body composition, sarcopenia, cachexia, myoesteatosis, malnutrition, sarcopenic obesity, computed tomography

## Abstract

There is increasing evidence supporting the use of morphofunctional assessment (MFA) as a tool for clinical characterization and decision-making in malnourished patients. MFA enables the diagnosis of malnutrition, sarcopenia, obesity, and cachexia, leading to a novel phenotype-based classification of nutritional disorders. Bioelectrical impedance analysis (BIVA), nutritional ultrasound^®^ (NU) and computed tomography (CT) are included, along with functional tests like the Timed Up and Go test (TUG). Myoesteatosis, detectable via CT, can occur independently from nutritional phenotypes and has been identified as a significant mortality predictor in idiophatic pulmonary fibrosis (IPF). Our aim is to analyze the prevalence and overlap of nutritional phenotypes in IPF and evaluate the prognostic value of myoesteatosis. Our bicenter cross-sectional study included 82 IPF patients (84.1% male and with a medium age of 71.1 ± 7.35 years). MFA was performed using BIVA, NU, CT at the T12 level (CT-T12), the handgrip strength (HGS) test, and the TUG. CT-T12 BC parameters were analyzed using FocusedON^®^ software, while statistical analyses were conducted with JAMOVI version 2.3.22. All four major nutritional phenotypes were represented in our cohort, with significant overlap. A total of 80.5% met the GLIM criteria for malnutrition, 14.6% had cachexia, 17% were sarcopenic, and 28% were obese. Of the obese patients, 70% were also malnourished, while 100% of sarcopenic obese patients (5.9% of total) had malnutrition. A total of 55% of sarcopenic patients with available CT also had myosteatosis, suggesting muscle quality deterioration as a potential driver of functional impairment. The presence of myosteatosis > 15% in T12-CT was an independent predictor of 12-month mortality (HR = 3.13; 95% CI: 1.01–9.70; *p* = 0.049), with survival rates of 78.1% vs. 96.6% in patients with vs. without myosteatosis, respectively. To conclude, this study underscores the relevance of MFA in the nutritional characterization of patients with IPF, demonstrating its potential to identify specific phenotypes associated with malnutrition, functional impairment, and the presence of **myoesteatosis,** thereby providing a valuable tool for clinical decision-making.

## 1. Introduction

Idiopathic pulmonary fibrosis (IPF) is a chronic and progressive lung disease of unknown etiology, characterized by interstitial fibrosis of the parenchyma, rapid and irreversible progression, and poor prognosis [1,2,3] despite the new antifibrotic medications [2] (mean survival time post-diagnosis of 5.5 ± 0.54 years in a cohort of 195 IPF patients [4]), whose estimated incidence appears to be increasing [2,3].

IPF constitutes a complex and multifactorial condition whose prognosis is also influenced by several comorbidities [5]. Chronic obstructive pulmonary disease (COPD) and gastroesophageal reflux disease [6] are considered frequent comorbidities in IPF [7], pulmonary hypertension stands out as a major prognostic factor [8], and emerging data underscore complex links with cardiovascular disorders [9]. Other conditions, such as diabetes, lung cancer, and arteriosclerosis, have also been extensively researched [1,10,11]. However, there remains limited literature on nutritional disorders [12,13,14], including malnutrition (defined with GLIM criteria) [12,15], whose presence in IPF patients is associated with all-cause hospitalization and mortality [16] and may serve as a prognostic indicator for antifibrotic therapy discontinuation [17]; sarcopenia (according to EWGSOP2 criteria) is characterized by low muscle strength, low muscle quantity or quality, and low physical performance [18,19] and associated with a higher incidence of disability and falls, poor quality of life, increased ICU length of stay [20], and mortality [18,21,22]. In IPF, a prevalence of sarcopenia at diagnosis of 23% [23] has been demonstrated and linked with higher severity [23] and lower median survival [4,24]. Cachexia (as defined by Evans’ criteria) [25] is a complex metabolic syndrome characterized by involuntary weight loss, including the loss of muscle and usually the loss of fat mass, resulting from a chronic inflammatory catabolic state that is associated with underlying diseases, such as chronic pulmonary disease, which cannot be fully reversed by conventional nutritional support [12,25,26,27,28]. Sarcopenic obesity, defined as a Body Mass Index (BMI) ≥ 30 combined with a diagnosis of sarcopenia according to EWGSOP2 criteria [12,19,29], not only includes the sarcopenic risks mentioned earlier but also involves the cardiovascular risks associated with obesity itself, which may even render patients ineligible for lung transplants [13,30]. Other conditions that involve changes in body composition as myosteatosis, characterized by fat accumulation within muscle tissue, promoting oxidative stress, insulin resistance, and inflammation [30,31], have been associated with an increased risk of severe COVID-19 via computed tomography at the T12 level (T12-CT) [32] and with the worsening of lung function loss in IPF [30].

All these diagnoses are interrelated [12,29]. Thus, myoesteatosis can manifest independently of sarcopenia, although in certain cases it may precede the onset of sarcopenia or exacerbate its consequences. Likewise, sarcopenia could be a component of cachexia, although it is not a prerequisite for its development, and malnutrition could be present despite all other nutritional-related diagnoses. These nutritional disorders are marked by changes in body composition [12,18,25,29,31], and some also by functional impairments [18,19,29]. Therefore, a comprehensive morphofunctional assessment (MFA) is required for their proper diagnosis [33,34], which includes body composition techniques like bioelectrical impedance analysis (BIVA), nutritional ultrasound^®^ (NU), and T12-CT, along with functional tests such as the handgrip strength (HGS) test and the Timed Up and Go test (TUG). Our study focuses on the advanced morphofunctional phenotyping of nutritional status to provide a more precise characterization of BC changes and their clinical implications in patients with IPF. By integrating novel imaging and functional assessment techniques, we aim to identify specific phenotypic patterns linked to malnutrition, sarcopenia, and myosteatosis. This innovative approach seeks to refine nutritional and therapeutic strategies, ultimately contributing to more personalized patient management and improving clinical outcomes in IPF.

## 2. Materials and Methods

### 2.1. Setting Study

This prospective, observational, bicenter study was conducted as part of routine clinical practice in the Nutrition Unit of Virgen de la Victoria University Hospital. The cohort included patients diagnosed with IPF at various stages. The majority of diagnoses were established during routine biannual evaluations conducted by the Pneumology Department of Virgen de la Victoria University Hospital, with additional participants recruited from the Regional University Hospital of Málaga. All participants provided written informed consent prior to enrollment. The study adhered to the ethical principles outlined in the Declaration of Helsinki and was approved by the Málaga Ethics Committee on 5 April 2022 (reference number 1743-N-21).

All individuals recruited for the present study met the specified inclusion criteria (the diagnosis of idiopathic pulmonary fibrosis, provision of informed consent for participation in the research, and completion of a computed tomography scan within a three-month period preceding or succeeding the initial nutritional assessment), while concurrently not satisfying any of the exclusion criteria (refusal to participate in the study; incapacity to undergo Bioelectrical Impedance Vector Analysis due to various factors such as ethnicity, significant dermatological conditions, fluid extravasation through the affected route, localized hematomas, amputation, etc.; or a prognosis of less than three months of life). The patient recruitment and selection processes are summarized in Figure S1.

Additionally, the cohort was compared with a healthy control group of 32 individuals with similar baseline characteristics recruited from the Nutrition Unit of Virgen de la Victoria University Hospital to evaluate changes in body composition related exclusively to aging, in order to better differentiate them from those related to IPF.

### 2.2. Body Composition Techniques (BIVA, NU, CT)

Body composition was assessed using the following methods: BIVA measurements were performed with a V 101 Whole Body Bioimpedance Vector Analyzer (AKERN, Florence, Italy) using tetrapolar electrodes placed on the right hand and foot in a supine position after a 5 min rest. Standardized phase angle (PhA) values were calculated using reference data from healthy Italian adults, adjusted for age and sex [35,36]. For NU, we conducted measurements with a 10–12 MHz probe and a multifrequency linear array (Mindray Z60, Madrid, Spain) of the rectus femoris quadriceps muscle, performing the ultrasonography in a supine position to obtain the rectus femoris axes (RF-Y-axis and RF-X-axis), circumference (RF-CIR), cross-sectional area (RF-CSA), and the subcutaneous fat of the leg (L-SAT); we also performed an abdominal adipose tissue assessment, measuring the midpoint between the xiphoid process and the navel to obtain T-SAT (total subcutaneous abdominal fat), S-SAT (superficial adipose subcutaneous abdominal fat), and VAT (preperitoneal or visceral fat). Finally, CT imaging at the T12 level (CT_T12) was conducted within three months of the initial nutritional assessment and analyzed using the FocusedON^®^ software, https://focusedon.es (accessed on 3 February 2025) [37,38,39]. We used an automatic tissue segmentation tool based on artificial intelligence that allowed for manual corrections if needed, developed by ARTIS Development and used in some FPI studies [39]. CT_T12 was chosen because it was close to L3 (the vertebrae level most used for body composition) and showed a good correlation in various studies [20,40,41,42], being especially useful for thoracic pathologies that were followed with thoracic CT where L1 or L2 were not present in many cases, and L3 in almost any cases [43,44]. The Hounsfield units (HUs) used for skeletal muscle mass were −29 to +150, −190 to −30 for subcutaneous adipose tissue (SAT) and intermuscular adipose tissue (IMAT) and −150 to −50 for visceral adipose tissue (VAT). The assessment of body composition using computed tomography at the T12 level focuses on analyzing different muscle groups compared to those evaluated at L3. At this level, the most relevant muscles include the paravertebral muscles (erector spinae, composed of the iliocostalis, longissimus, and spinalis) and the quadratus lumborum, as well as the abdominal wall muscles (external oblique, internal oblique, transversus abdominis, and rectus abdominis). Additionally, at higher sections of T12, the dorsal muscles, such as the latissimus dorsi, serratus posterior inferior, and intercostal muscles, can be observed, playing a crucial role in respiratory function. Although the psoas muscle is more prominently visualized at lower levels, like L3, its origin may be identified in some cases at T12.

After evaluating the skeletal muscles mentioned above, we obtained the T12 Skeletal Muscle Area (SMA_T12CT, cm^2^ and %), and, by incorporating patients’ heights, we calculated the T12 muscle index (SMI_T12CT, cm^2^/m^2^). Moreover, we analyzed the intramuscular adipose tissue area (IMAT, cm^2^ and %), the subcutaneous fat area (SFA) (cm^2^ and %), and the visceral fat area (VFA) (cm^2^ and %).

### 2.3. Functional Assessment (HGS, TUG, SPIROMETRY, DLCO)

Functional assessments included HGS testing, which comprised measuring grip strength when seated with a 90° position of the dominant arm through three maximal isometric contractions with a JAMAR hand dynamometer (Asimow Engineering Co., Los Angeles, CA, USA) to obtain the median value, used for screening of sarcopenia, and the TUG, which measured the number of seconds required to stand up from a wheelchair, walk three meters, turn round to the chair, and sit back down again to assess functional capacity. Moreover, all patients underwent spirometry, lung volume, and diffusion capacity testing at the Pulmonary Function Unit.

### 2.4. Nutritional Phenotypes Proposed (Cachexia, Malnutrition, Sarcopenia, Obesity, and Sarcopenic Obesity)

Cachexia, considered as a synonym for chronic disease-related malnutrition (DRE) with inflammation [12], is defined according to Evans’ criteria [25] as involuntary weight loss ≥ 5% in the past 12 months (or BMI < 20) and at least three of the following: anorexia (intake < 70% of requirements), low muscle mass (measured in this study as a low ASMI—appendicular skeletal muscle mass index—or ASMM—appendicular skeletal muscle mass—<5.45 kg/m^2^ (women)/<7.25 kg/m^2^ (men)), and elevated biomarkers (CRP > 5 mg/L).

Myoesteatosis has also been considered in other definitions of cachexia, such as Martin’s criteria [14], which include a weight loss > 5% in 6 months, myoesteatosis, and sarcopenia diagnosed by lumbar CT. For our study, we defined myoesteatosis using the following formula: IMAT (cm^2^)/(SMA (cm^2^) + IMAT (cm^2^)) × 100, with myoesteatosis defined as IMAT > 10%, as suggested by reference [43].

Malnutrition (referring to DRE) was diagnosed using GLIM criteria [15], requiring one phenotypic criterion (e.g., weight loss > 5% in 6 months, BMI < 20 if <70 years old, or low muscle mass, measured in this study as a low ASMI/ASMM) and one etiological criterion (in our case, the diagnosis of IPF). Severity was classified as moderate (≥5% weight loss in 6 months, BMI < 20 if <70 years or <22 if ≥70 years) or severe (≥10% weight loss, BMI < 18.5 if <70 years or BMI < 20 if ≥70 years.

Sarcopenia was diagnosed per the EWGSOP2 criteria [19]: probable sarcopenia was identified by low HGS (<27 kg for men; <16 kg for women), confirmed sarcopenia by low HGS plus low ASMI (<7.0 kg/m^2^ for men; <5.5 kg/m^2^ for women), and severe sarcopenia by the additional presence of low physical performance (e.g., poor results on the TUG).

Obesity was defined based on the World Health Organization (WHO) criteria as a BMI ≥ 30 kg/m^2^ [44]. Sarcopenic obesity was classified using the ESPEN and EASO criteria [29]. This included the presence of obesity, defined as a high fat mass (FM), combined with sarcopenia. High FM was defined based on the following age- and sex-specific cut-off points: 20–39 years: >39% for females, >26% for males; 40–59 years: >41% for females, >29% for males (Caucasians); 60–79 years: >43% for females, >31% for males (Caucasians). Sarcopenia was determined based on low handgrip strength (<27 kg for men and <16 kg for women) and low skeletal muscle mass (SMM/kg), with cut-off points of <37% for men and <27.6% for women, as proposed by the ESPEN/EASO consensus.

### 2.5. Statistical Analysis

Descriptive statistical analyses were conducted to characterize the sociodemographic and clinical variables of the study population. Continuous variables with normal distributions were expressed as means ± standard deviations, whereas categorical variables were presented as percentages. Differences between groups, such as sex, nutritional phenotypes, or health status (IPF vs. controls), were assessed using the Mann–Whitney U test for non-parametric data and Student’s *t*-test for parametric variables, following the confirmation of normality using the Shapiro–Wilk test.

Receiver Operating Characteristic (ROC) curves were constructed to determine the optimal cut-off points for identifying myosteatosis, maximizing diagnostic accuracy via the Youden index. Diagnostic performance was evaluated by calculating the Area Under the Curve (AUC), sensitivity, and specificity for each identified cut-off point.

Survival analysis was performed using Kaplan–Meier curves to evaluate differences in mortality risk between groups categorized by the presence or absence of myosteatosis. The significance of the differences in survival between the groups was determined using the log-rank test. Additionally, a multivariable Cox regression model adjusted for age, sex, and BMI was used to identify independent predictors of mortality, providing hazard ratios (HRs) with corresponding 95% confidence intervals (CIs).

All the statistical tests were two-sided, and the significance threshold was set at *p* < 0.05. Statistical analyses were conducted using JAMOVI software (version 2.3.28 for macOS).

## 3. Results

The study cohort consisted of 82 participants, predominantly male (84.1%) with a mean age of 71.1 ± 7.35 years. The men had a mean height of 159.0 ± 4.8 cm and a mean BMI of 30.1 ± 2.7, while the women had a mean height of 170.9 ± 7.0 cm and a mean BMI of 27.3 ± 3.4. Pulmonary function tests revealed moderate impairment, including a mean DLCO of 49.4 ± 17.6 L (n = 60) and an FVC% of 66.0 ± 16.6%. Comorbidities were common, with hypertension observed in 60.6% of participants and diabetes mellitus in 31.0%.

Quality of life was significantly reduced, as evidenced by a Saint George Total score of 42.8 ± 25.2 and an SF-12 Physical score of 37.7 ± 10.5, reflecting significant physical limitations. In addition, 35.3% of patients had respiratory insufficiency, and the overall mortality rate during the study period was 28.0%.

### 3.1. Morphofunctional Parameters

#### 3.1.1. Morphofunctional Differences by Sex

The baseline morphofunctional parameters of the study population are described in Table 1. Males had higher fat-free mass (56.9 ± 6.6 vs. 45.7 ± 3.2 kg), body cell mass (27.0 ± 4.9 vs. 20.7 ± 2.4 kg), and handgrip strength (35.7 ± 8.4 vs. 19.2 ± 4.9 kg), while females showed higher fat mass (30.7 ± 8.0 vs. 23.0 ± 7.5 kg) and leg subcutaneous adipose tissue (L-SAT: 1.7 ± 0.6 vs. 0.6 ± 0.3 cm). Functional differences were also observed, with males performing better in the TUG (7.3 ± 1.8 vs. 9.9 ± 2.7 s) and 6MWT (412.8 ± 63.8 vs. 375.1 ± 64.1 m). These differences reflect variations in body composition and functional metrics between males and females.

#### 3.1.2. Morphofunctional Differences Between IPF Patients and Healthy Controls

Significant differences in morphofunctional parameters between IPF patients and healthy controls can be observed in Table 2. IPF patients exhibited lower Pha and reactance, Xc, indicative of impaired cell-membrane integrity and reduced muscle quality. The negative Spha observed in the IPF group reflects their deviation below expected values for age, sex, and BMI, consistent with the impact of the disease on tissue composition and catabolic balance. Nutritional ultrasound revealed smaller RF-CSA and RF-Y-axis measurements in the IPF group, reflecting pronounced muscle atrophy. These findings were accompanied by altered hydration percentages, highlighting systemic changes in body composition linked to IPF. Functionally, IPF patients demonstrated longer TUG times, signifying impaired mobility and physical performance. These results underscore the importance of advanced morphofunctional assessments, such as BIVA and NU, in evaluating the clinical impact of IPF.

### 3.2. Nutritional Phenotypes

We summarize the prevalence of phenotypes according to different diagnostic criteria in Table 3. For cachexia, the Evans criteria identified 13.4% of patients with cachexia. Using GLIM criteria, all patients met the etiological criterion due to their underlying IPF, with moderate and severe malnutrition observed in 71.8% and 9.4% of cases, respectively. Sarcopenia was identified in 16.5% of the population, with low muscle mass (low ASMI/ASMM) in 64.7% and dinapenia in 22.4%. Regarding obesity, our results indicated that 28% of the cohort was obese according to the criterion of a BMI ≥ 30. Sarcopenic obesity, affecting only 5.9% of patients, highlights the complex interplay between obesity and muscle mass in this population.

**Figure 1:**
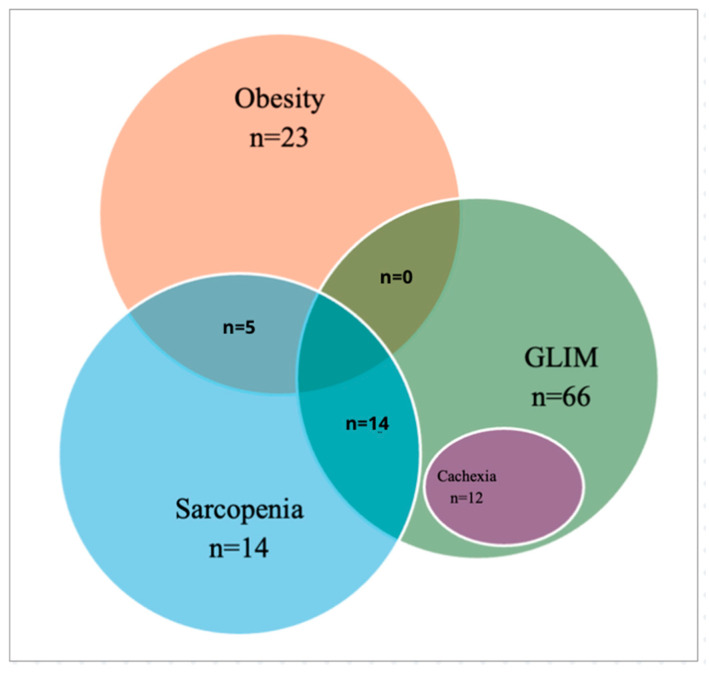
Nutritional phenotypes and their overlap. Abbreviations: Global Leadership Initiative on Malnutrion (GLIM) criteria.

The distribution and overlap of nutritional phenotypes in IPF patients illustrate their complexity and interrelation (Figure 1). They highlight the coexistence of GLIM-defined malnutrition, sarcopenia, obesity, sarcopenic obesity, and cachexia, demonstrating that many patients exhibit multiple nutritional disorders simultaneously. The high prevalence of malnutrition among obese and sarcopenic patients underscores the limitations of BMI-based assessments and the need for comprehensive morphofunctional evaluation.

Patients with cachexia, sarcopenia, and severe GLIM malnutrition exhibited significant reductions in key morphofunctional parameters (Table 4). Compared to their respective non-affected groups, these patients had a lower fat-free mass (FFM), body cell mass (BCM), rectus femoris cross-sectional area (RF-CSA), and muscle area at T12. Functional impairment was also evident, with reduced handgrip strength (HGS) and, notably, poorer performances in the six-minute walk test (6MWT) and timed up-and-go test (TUG) in sarcopenic patients.

Significant differences can be observed, in Table 5, between the non-obese and obese groups, with obese individuals showing better cellular integrity, higher fat-free mass, and greater muscle area, but alterations in fluid balance. In contrast, obese sarcopenic individuals had lower muscle quality, reduced handgrip strength, slower mobility, and diminished regional muscle measurements compared to the non-sarcopenic patients.

Significant differences in fat-related variables were observed between the groups. Table 6 shows that obese individuals exhibited a higher fat mass index (FMI) and subcutaneous adipose tissue area (SAT_area_T12) compared to non-obese individuals, reflecting greater fat deposition. Additionally, SAT density (SAT_HU_T12) was significantly lower in obese individuals, indicating differences in fat composition. Among obese sarcopenic individuals, total subcutaneous adipose tissue (T-SAT) was significantly greater, suggesting alterations in fat distribution compared to their non-sarcopenic counterparts. However, no significant differences were observed in visceral adipose tissue (VAT) parameters across groups.

**Figure 2:**
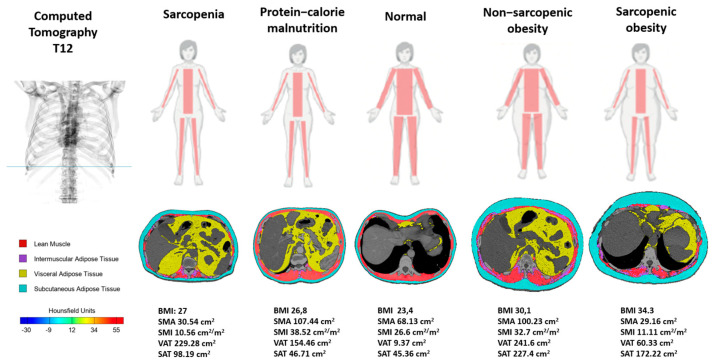
Nutritional phenotypes assessed by T12-CT. Abbreviations: BMI: Body Mass Index (kg/m^2^); SMA: Skeletal Muscle Area (cm^2^); SMI: Skeletal Muscle Index (cm^2^/m^2^); VAT: visceral adipose tissue (cm^2^); SAT: subcutaneous adipose Tissue (cm^2^).

Based on the CT scans, we have visually represented the distinct phenotypes identified (Figure 2) within the sample of patients with idiopathic pulmonary fibrosis (IPF). This includes showcasing key variations in body composition and their corresponding clinical characteristics, enabling a clearer understanding of the phenotypic diversity present in this population.

### 3.3. Determination of Cut-Off Point Value and Prognostic Analysis for Myoesteatosis

**Figure 3:**
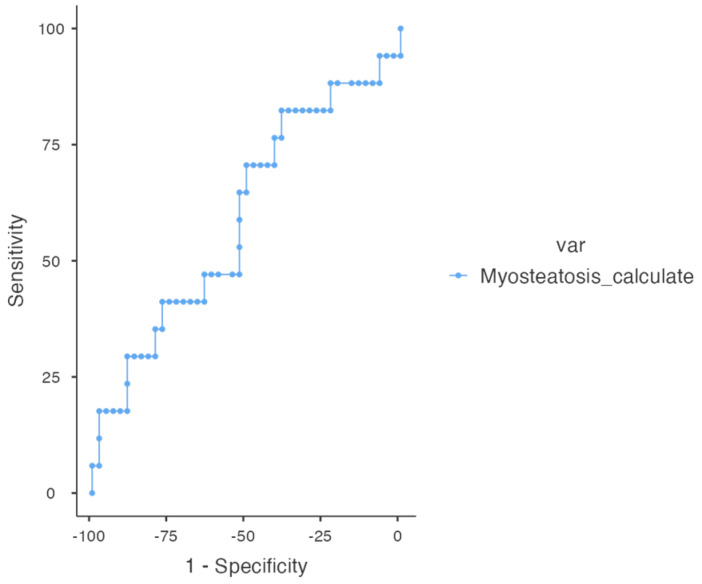
ROC curve cut-off point for myosteatosis.

The analysis to determine the optimal cut-off point for myosteatosis, conducted exclusively in men due to the low representation of women in the dataset, identified 15.25% as the most effective threshold for predicting mortality (Figure 3). This cut-off demonstrated the highest Youden index (0.317), reflecting a well-balanced trade-off between sensitivity (70.59%) and specificity (61.11%). Additionally, the Area Under the Curve (AUC) was 0.663, indicating a moderate ability to distinguish between survival and mortality.

The presence of myosteatosis was significantly associated with a higher risk of adverse events (mortality) (HR = 3.13; 95% CI: 1.01–9.70; *p* = 0.049) compared to patients without myosteatosis (Table 7). At 12 months, survival was lower in the myosteatosis group (78.1%; 95% CI: 65.0–93.8%) compared to the group without myosteatosis (96.6%; 95% CI: 90.1–100.0%) (Table 7). These findings highlight that myosteatosis is a relevant prognostic marker, associated with an increased risk of mortality and reduced short-term survival, emphasizing its importance in risk stratification for this patient population.

**Figure 4:**
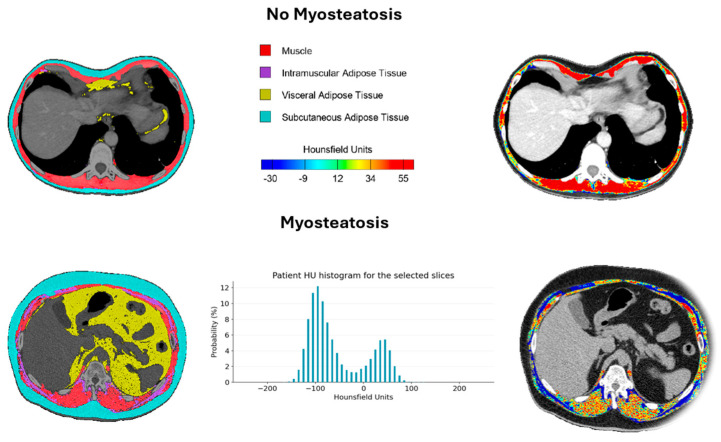
Representation of myosteatosis by Hounsfield units measured by CT12.

We can distinguish [43,44] two distinct phenotypes based on intramuscular fat content: no myosteatosis and myosteatosis. In the no myosteatosis phenotype, the CT scans show muscle tissue with minimal fat infiltration, characterized by higher Hounsfield unit (HU) values, indicative of healthy musculature. In contrast, the myosteatosis phenotype demonstrates significant intramuscular fat accumulation, quantified as 15% myosteatosis, with corresponding lower HU values in the affected muscle tissue (Figure 4).

### 3.4. Survival Analysis Based on the Myosteatosis Cut-Off Point

To evaluate the prognostic value of myosteatosis in the patients, Cox regression analysis was performed to calculate the risk of adverse events (mortality), with the model adjusted for age, sex, and BMI. The presence of myosteatosis was determined using the formula described in the Materials and Methods section. From the total sample, 61 patients had an available CT scan performed approximately 3 months prior to their nutritional assessment.

**Figure 5:**
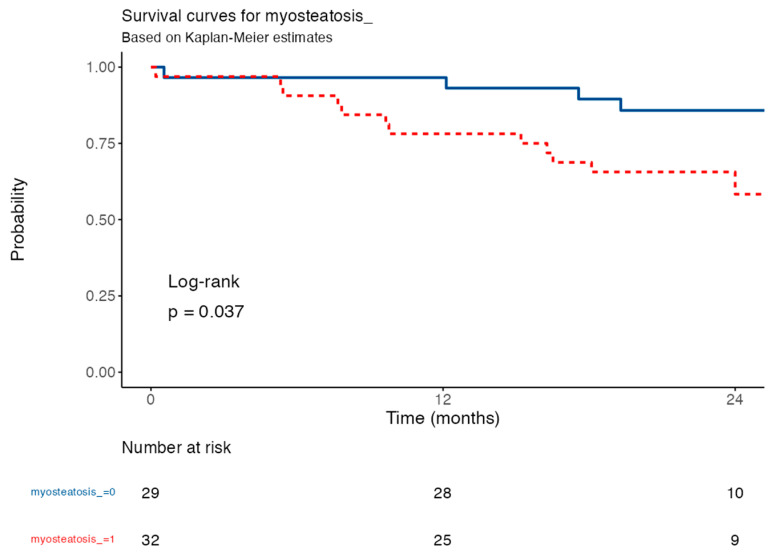
Kaplan–Meier survival curve of IPF patients with myoesteaosis. IPF patients with myoesteatosis (red line) died more frequently than those with non-myoesteatosis (blue line), as measured by CT, over a 24-month follow-up period.

The Kaplan–Meier survival curve (Figure 5) shows that IPF patients with myoesteatosis (red line) had higher mortality rates compared to those without (blue line) over 24 months. These findings indicate that myoesteatosis is an important prognostic marker, as its presence correlates with reduced survival, emphasizing its role in risk stratification for IPF patients.

To sum up, our data revealed that 80.5% of patients met the GLIM criteria for malnutrition (*p* < 0.001), 14.6% fulfilled Evans’ cachexia criteria (*p* < 0.05), and 17.0% met the European Working Group on Sarcopenia in Older People (EWGSOP2)’s definition of sarcopenia (*p* < 0.05). Obesity was present in 28.0% of participants (*p* < 0.01), of whom 70.0% were concurrently malnourished (*p* < 0.01). Notably, patients with myosteatosis greater than 15% in T12-CT had an increased 12-month mortality risk (HR = 3.13; 95% CI: 1.01–9.70; *p* = 0.049), with a survival rate of 78.1% versus 96.6% among those without myosteatosis. Furthermore, sarcopenic patients exhibited significantly lower mean handgrip strength (19.7 ± 4.8 kg vs. 35.4 ± 8.8 kg, *p* < 0.001) and a longer Timed Up and Go test duration (8.9 ± 2.3 s vs. 7.5 ± 2.1 s, *p* < 0.05) than their non-sarcopenic counterparts. Collectively, these results underscore the clinical importance of each phenotype and highlight the robust associations between morphofunctional parameters and key outcomes.

## 4. Discussion

To our knowledge, this is the first study to incorporate a comprehensive morphofunctional assessment (including BIVA, NU and T12-CT) alongside functional tests in order to phenotype nutritional diagnoses (malnutrition, sarcopenia, obesity, sarcopenic obesity, and cachexia) in IPF patients. Our work focuses on the advanced morphofunctional phenotyping of nutritional status in IPF, aiming to better characterize BC changes, clarify their clinical implications, and, ultimately, pave the way for more personalized patient management.

Our cohort was composed of 82 patients (84.1% men), with a mean age at diagnosis of 71.1 ± 7.35 years, consistent with findings reported in the literature [1]. Over half (51,1%) were at GAP stage 2, and 26.7% at stage 3, with a 28.0% mortality rate at one-year follow-up, aligning with previous reports [2,4]. Despite antifibrotic therapy (98.6% of our patients), participants exhibited markedly reduced qualities of life and significant physical limitations, as described in other studies [45].

BC analysis confirmed well-documented biological differences between the sexes in our cohort, as males had significantly higher FFM and BCM, but less FM and L-SAT compared to females. Moreover, BC analysis allowed for a better evaluation of the impact of IPF on BC. As shown in Table 2, patients with IPF demonstrated impaired cell-membrane integrity, as evidenced by their reduced PhA and Xc, as well as significant muscle quality and quantity deterioration compared to healthy controls.

Likewise, the time required to perform the TUG was significantly higher in FPI patients, particularly in female patients, suggesting potential sex-specific vulnerabilities that should be further studied in future investigations.

MFA [28,29] integrating data from body composition and functionality could potentially enhance our understanding of nutritional diagnosis. Our study introduces five proposed nutritional phenotypes—GLIM-defined malnutrition, sarcopenia, obesity, sarcopenic obesity, and cachexia—that could serve as a framework for personalized nutritional management. Each phenotype has been linked to poorer outcomes, underscoring the clinical relevance of targeted interventions.

First, malnutrition. This is well-documented among IPF patients [13,14,46,47] and often correlates with worse prognoses and higher mortality rates [16,17,46]. Unlike general nutritional risk assessments, using tools such as NRS-2002 [48], MUST [49] or MNA [50], GLIM criteria [12,15] provide a more specific diagnosis of disease-related malnutrition. In our cohort, malnutrition by GLIM criteria was present in 80.5% of patients, making it the most frequent phenotype.

Limited research has addressed other nutritional diagnoses such as sarcopenia or cachexia in patients with IPF, despite the growing interest in these topics.

Cachexia, according to Evans’ criteria [25], was identified in 13.4% of our patients. Notably, 100% of the cachectic patients also had malnutrition, which aligns with the proper definition of cachexia as chronic disease-related malnutrition requiring multidisciplinary management, as conventional nutritional support alone does not fully reverse it [12,25,26,27,28]. Although the literature on cachexia in IPF patients is scarce, studies on COPD, such as one conducted on a sample of 1,446,431 COPD patients, have demonstrated its association with increased inpatient mortality, higher resource utilization, and prolonged hospital stays in the 115,276 patients (8% of the sample) who met the criteria for cachexia [51].

Sarcopenia, defined according to EWGSOP2 criteria [18,19], has been linked in IPF to higher illness severity [23] and lower median survival [4,24]. Faverio et al. reported a sarcopenia prevalence of 23% at IPF diagnosis in a cohort of 83 patients [23]. In our sample, 16.5% of patients were diagnosed with sarcopenia, even though 64.7% already had a low muscle mass. We adhered to the thresholds recommended for BIVA [19], though alternative cut-off values for IPF patients using different body composition techniques have been proposed. For instance, low muscle mass cut-offs of have been suggested for IPF patients at RF-CSA < 3.00 cm^2^ in NU [52] and SMI_T12CT < 28.8 cm^2^/m^2^ in T12-CT [39]. However, these cut-off values require further validation due to the limited sample sizes in their supporting studies. T12 is preferred for thoracic pathologies such as IPF, given that thoracic CT scans are routinely used for follow-up assessments [39,42,53,54]. Moreover, muscle area in T12-CT has been shown to predict both short- and long-term survival in IPF patients [4]. The T12 cut-off values proposed by Alahmad et al. were 42.6 cm^2^/m^2^ for men and 30.6 cm^2^/m^2^ for women, determined through linear regression analysis based on the validated SMI values at L3, and patients with lower SMI_T12CT exhibited prolonged hospital stays.

Finally, obesity, traditionally defined as BMI ≥ 30 [12], was present in 28% of our sample. Nevertheless, BMI alone is increasingly being deprioritized in the diagnosis of obesity in favor of body composition metrics such as FM percentage standardized by height in BIVA [29] and/or waist circumference [55,56]. A recent review by Donini et al. highlights the urgent need to use body composition techniques for obesity and sarcopenic obesity characterization [56]. In our cohort, 40.2% of patients had a high FM in BIVA [49]. Additionally, 70% of our patients with obesity by OMS criteria and 100% of the sarcopenic obese patients according to EASO/ESPEN criteria were malnourished.

A study with a similar initiative to ours, though lacking NU or T12-CT assessments, evaluated 90 IPF patients with an age and GAP-stage distribution comparable to our cohort. It found that 67.8% of patients had a normal nutritional status, 14.9% were at risk of malnutrition (assessed using MNA but not GLIM criteria), 25.3% had non-sarcopenic obesity, 4.6% had sarcopenia, and 2.3% had sarcopenic obesity. Notably, no cases of cachexia were identified, but the Evans criteria were not applied [57]. While the study provides valuable insights, it does not utilize comprehensive diagnostic frameworks such as the GLIM criteria for malnutrition or the Evans criteria for cachexia. In contrast, our study adopts a more detailed and integrative approach, incorporating advanced morphofunctional assessments, including NU and T12-CT, to better capture the complexity of nutritional phenotypes in IPF.

To conclude, all these diagnoses are interrelated and yet could exist independently [12,29] (as illustrated in Figure 1), including myoesteatosis (although it is not included in Figure 1). Among a subset of 61 patients who underwent T12-CT body composition analysis, 55% of sarcopenic patients also had myoesteatosis.

We introduce the concept of myosteatosis as an independent entity, even though it does not fulfill the requirements for a nutritional phenotype. It is characterized by fat accumulation within muscle tissue and can be assessed using CT [30,31]. Myoestatosis has been linked to the worsening of lung function loss in IPF [30]. In a cohort of 79 IPF patients with a mean age of 70 years, a median survival of 35 months was reported for those with a myosteatosis index below the sample median, compared to 14 months for those above the median (*p* = 0.0056) [43]. An IMAT > 10% in T12-CT was the threshold associated with higher mortality, identifying patients with significantly reduced survival rates [43]. Additionally, the myoesteatosis index, defined as the percentage of intramuscular and intermuscular fat relative to total body fat (19.45% ± 3.88% in patients surviving < 2 years vs. 16.29% ± 3.33% in those surviving > 2 years, *p* < 0.001), was identified as an independent predictor of mortality in a multivariate analysis (HR = 1.12, *p* = 0.005) (45). Based on these findings, we propose that myoesteatosis be considered an independent mortality factor in this patient population, without the need for inclusion in existing nutritional diagnostic frameworks. Nonetheless, our study found IMAT ≥ 15% to be an independent mortality risk factor, and Salhöfer et al. reported significance at 10%. We attribute this discrepancy to our smaller sample size (61 patients with CT performed in routine clinical practice). Routine CT scans in clinical practice may provide additional insights into muscle quality, a parameter not as effectively captured by BIVA or NU.

Nutritional phenotyping is an urgent clinical need, as differentiating malnutrition, sarcopenia, cachexia, obesity, and sarcopenic obesity, or their co-occurrence, has practical implications. Patients meeting GLIM criteria require a gradual increase in caloric and protein intake (27–30 kcal/kg/day and 1.2–1.5 g/kg/day), with close monitoring in cases at risk of refeeding syndrome [58,59]. However, managing obesity focuses on an hypocaloric, protein-rich diet combined with aerobic and resistance exercise, plus potential anti-obesity pharmacological agents, or bariatric surgery in severe cases [56,58]. In contrast, the management of sarcopenic obesity prioritizes muscle preservation through a normocaloric, high-protein diet and progressive resistance training [56,58]. Advanced MFA thus emerges as a central tool for tailoring interventions to each patient’s phenotype.

Our study presents several limitations worth noting. Firstly, the relatively small sample size for specific nutritional phenotypes, such as sarcopenic obesity or cachexia, may limit our ability to identify significant associations. Additionally, the predominance of male participants (84.1%) restricts the generalizability of our findings, particularly concerning sex-specific differences. Moreover, the reliance on routine T12-CT scans, available for only 61 patients, may have reduced the statistical power of certain analyses. Lastly, the cross-sectional design does not allow for the capturing of longitudinal changes or the evaluation of causal relationships. Despite these limitations, our study is innovative, as it integrates advanced morphofunctional assessment techniques to characterize nutritional phenotypes in idiopathic pulmonary fibrosis.

Future research should focus on longitudinal studies to track the evolution of nutritional phenotypes and their clinical impacts. Balanced gender representation is essential to explore sex-specific vulnerabilities. Validating cut-offs for sarcopenia, myosteatosis, and other parameters in larger multicenter cohorts will strengthen clinical utility. Investigating tailored interventions, including personalized diets, exercise programs, and pharmacological strategies, will provide valuable insights. Finally, exploring the prognostic role of myosteatosis and its integration into routine assessments may enhance nutritional diagnosis and management.

## 5. Conclusions

Our study highlights the critical role of MFA in identifying distinct nutritional phenotypes in IPF. The high prevalence and overlap of malnutrition, sarcopenia, obesity, and cachexia underscore the limitations of traditional nutritional screening methods and emphasize the need for a phenotype-based approach. Importantly, myosteatosis, assessed through CT at the T12 level, emerged as an independent predictor of mortality, reinforcing its potential value in risk stratification. These findings advocate for the routine integration of advanced body composition techniques, such as BIVA, NU, and CT, into clinical practice in order to enable personalized nutritional and therapeutic strategies. Future research should focus on validating these phenotypes in larger cohorts and exploring targeted interventions to improve patient outcomes. 

## Figures and Tables

**Table 1:** Morphofunctional parameters measured at baseline by sex.

Variables	Females (n = 13)	Males (n = 69)	*p*-Value
Weight (kg)	76.4 ± 9.3	79.9 ± 12.7	0.341
Height (cm)	159.0 ± 4.8	170.9 ± 7.0	<0.001
BMI (kg/m^2^)	30.1 ± 2.7	27.3 ± 3.4	0.004
BIVA Parameters			
Rz (Ω)	562.7 ± 62.7	515.5 ± 60.2	0.029
Xc (Ω)	44.5 ± 5.7	44.1 ± 8.3	0.859
FFM (kg)	45.7 ± 3.2	56.9 ± 6.6	<0.001
BCM (kg)	20.7 ± 2.4	27.0 ± 4.9	<0.001
TBW (L)	34.0 ± 2.5	42.5 ± 5.4	<0.001
FM (kg)	30.7 ± 8.0	23.0 ± 7.5	0.003
Pha (°)	4.5 ± 0.5	4.9 ± 0.8	0.097
NAK (ratio)	1.08 ± 0.11	1.18 ± 0.19	0.089
Hydration (%)	74.3 ± 1.9	74.7 ± 2.4	0.482
Nutrition (score)	624.0 ± 73.2	791.1 ± 162.1	<0.001
Nutritional Ultrasound (NU)			
RF-CSA (cm^2^)	2.4 ± 0.4	3.6 ± 1.0	<0.001
RF-Y-Axis (cm)	1.0 ± 0.2	1.1 ± 0.3	0.026
L-SAT (cm)	1.7 ± 0.6	0.6 ± 0.3	<0.001
T-SAT (cm)	2.4 ± 0.7	1.6 ± 0.7	<0.001
S-SAT (cm)	1.1 ± 0.3	0.7 ± 0.2	<0.001
VAT (cm^2^)	0.8 ± 0.5	0.6 ± 0.3	0.043
Functional Tests			
HGS (kg)	19.2 ± 4.9	35.7 ± 8.4	<0.001
TUG (s)	9.9 ± 2.7	7.3 ± 1.8	<0.001

Mann–Whitney U test (MWU) used for non-normal distributions (Shapiro–Wilk *p* < 0.05). Student’s *t*-test applied for comparisons with normal distributions (Shapiro–Wilk *p* > 0.05). Values reported as means ± standard deviations for each group, with *p*-values indicating significant differences where applicable. Abbreviations: phase angle (Pha); reactance (Xc); rectus femoris cross-sectional area (RF-CSA); rectus femoris Y-axis thickness (RF-Y-Axis); fat mass (FM); Timed Up-and-Go test (TUG); Bioelectrical Impedance Vector Analysis (BIVA); fat-free mass (FFM); body cell mass (BCM); Total Body Water (TBW); Sodium-to-Potassium Ratio (NAK); nutritional ultrasound (NU); Body Mass Index (BMI); Resistance (Rz); Leg subcutaneous adipose tissue (L-SAT), Superficial abdominal adipose tissue (S-SAT), Total abdominal adipose tissue (T-SAT), Hand grip strength (HGS).

**Table 2:** Comparison of morphofunctional parameters between FPI patients and control group.

Variable	IPF (Mean ± SD)	Control (Mean ± SD)	*p*-Value
Patients (n)	82	32	
Gender (Male)	84.1% (69/82)	68.75% (22/32)	*p* = 0.66
Age	71.1 ± 7.35	70.2 ± 5.19	*p* = 0.526
Weight (kg)	79.39 ± 12.28	72.98 ± 13.21	0.016
Height (cm)	169.05 ± 7.98	165.91 ± 9.12	0.072
BMI (kg/m^2^)	27.73 ± 3.45	26.35 ± 3.29	0.054
BIVA Parameters			
Rz (Ω)	522.96 ± 62.65	520.47 ± 84.74	0.352
Xc (Ω)	44.18 ± 7.92	49.86 ± 5.12	<0.001
FFM (kg)	55.17 ± 7.45	53.92 ± 10.36	0.940
BCM (kg)	26.03 ± 5.11	27.82 ± 6.51	0.124
TBW (L)	41.19 ± 5.95	39.62 ± 7.67	0.247
FM (kg)	24.22 ± 8.05	19.05 ± 5.66	0.001
Pha (°)	4.85 ± 0.77	5.54 ± 0.61	<0.001
NAK (ratio)	1.16 ± 0.19	1.07 ± 0.08	0.009
Hydration (%)	74.61 ± 2.36	73.44 ± 0.23	0.006
Nutrition (score)	764.61 ± 163.19	832.97 ± 183.75	0.055
Spha (°)	−0.92 ± 0.94	−0.26 ± 0.48	<0.001
Nutritional Ultrasound (NU)			
RF-CSA (cm^2^)	3.37 ± 1.00	4.58 ± 1.61	<0.001
RF-Y-Axis (cm)	1.12 ± 0.28	1.40 ± 0.34	<0.001
L-SAT (cm)	0.81 ± 0.53	0.83 ± 0.46	0.845
T-SAT (cm)	1.70 ± 0.73	1.60 ± 0.64	0.498
S-SAT (cm)	0.74 ± 0.30	0.70 ± 0.30	0.538
VAT (cm)	0.66 ± 0.30	0.66 ± 0.28	0.907
Functional Tests			
HGS (kg)	33.11 ± 10.02	32.56 ± 8.03	0.785
TUG (s)	7.72 ± 2.17	5.79 ± 1.12	<0.001

Mann–Whitney U test (MWU) used for non-normal distributions (Shapiro–Wilk *p* < 0.05). Student’s *t*-test applied for comparisons with normal distributions (Shapiro–Wilk *p* > 0.05). Values reported as means ± standard deviations for each group, with *p*-values indicating significant differences where applicable. Abbreviations: Idiopathic pulmonary fibrosis (IPF); phase angle (Pha); reactance (Xc); standardized phase angle (Spha); rectus femoris cross-sectional area (RF-CSA); rectus femoris Y-axis thickness (RF-Y-Axis); fat mass (FM); Timed Up-and-Go test (TUG); Bioelectrical Impedance Vector Analysis (BIVA); fat-free mass (FFM); body cell mass (BCM); Total Body Water (TBW); Sodium-to-Potassium Ratio (NAK); nutritional ultrasound (NU); Body Mass Index (BMI); Resistance (Rz); Leg subcutaneous adipose tissue (L-SAT), Superficial abdominal adipose tissue (S-SAT), Total abdominal adipose tissue (T-SAT), Hand grip strength (HGS).

**Table 3:** Comprehensive table of nutritional phenotypes according to different criteria.

Nutritional Phenotype	Criteria	Counts (n)	% of Total
Cachexia, Evans’ Criteria		11	13.4%
	GLIM Cachexia	44	53.7%
	Low-Intake Cachexia	46	56.1%
	Inflammation Cachexia (CRP)	32	39.0%
	Low Muscle Mass (Low ASMI/ASMM)	52	63.4%
Sarcopenia, EWGSOP2 Criteria		14	16.5%
	Low Muscle Mass (Low ASMI/ASMM)	55	64.7%
	Dinapenia	19	22.4%
	Dysfunctionality (Severe Sarcopenia)	0	0%
Disease-Related Malnutrition, GLIM Criteria			
	No GLIM Malnutrition	16	18.8%
	Moderate Malnutrition	61	71.8%
	Lost Weight (Moderate)	21	25.6%
	BMI (Moderate)	23	28.0%
	Low Muscle Mass (Low ASMI/ASMM)	52	63.4%
	Severe Malnutrition	8	9.4%
	Lost Weight (Severe)	22	26.8%
	Low Muscle Mass (Low ASMI/ASMM)	52	63.4%
	BMI (Severe)	0	0.0%
Obesity and Sarcopenic Obesity			
	Obesity, WHO Criteria (BMI ≥ 30)	23	28.0%
	Sarcopenic Obesity, ESPEN/EASO Criteria	5.79 ± 1.12	<0.001
	Dinapenia	19	22.4%
	High Fat Mass (%)	33	40.2%
	SMM/KG	35	41.2%
	Dysfunctionality (Severe Sarcopenia)	0	0%

Abbreviations: GLIM (Global Leadership Initiative on Malnutrition); muscle and fat parameters include CRP (C-reactive protein), BMI (Body Mass Index), ESPEN (European Society for Clinical Nutrition and Metabolism), and EASO (European Association for the Study of Obesity), appendicular skeletal muscle index (ASMI), appendicular skeletal muscle mass (ASMM), World health organization (WHO), Skeletal muscle mass/kilogram (SMM/KG).

**Table 4:** Morphofunctional parameters by cachexia, sarcopenia, and severe GLIM malnutrition.

Parameter	Non-Cachexia (n = 65)	Cachexia (n = 17)	*p*	Non-Sarcopenia (n = 70)	Sarcopenia (n = 12)	*p*	Non-Malnutrition (n = 16)	Malnutrition, GLIM (n = 69)	*p*
Phase Angle (Pha)	4.91 ± 0.73	4.61 ± 0.89	0.173	4.90 ± 0.73	4.53 ± 0.93	0.156	5.17 ± 0.86	4.75 ± 0.72	0.046
Standardized Phase Angle (Spha)	−0.87 ± 0.96	−1.11 ± 0.88	0.397	−0.91 ± 0.91	−0.96 ± 1.15	0.572	−0.75 ± 1.05	−1.01 ± 0.93	0.335
Fat-Free Mass (FFM, kg)	56.41 ± 7.55	50.45 ± 4.79	0.002	56.24 ± 7.27	48.93 ± 5.20	0.001	61.04 ± 5.03	53.59 ± 7.32	<0.001
Body Cell Mass (BCM, kg)	26.84 ± 5.06	22.91 ± 4.09	0.007	26.72 ± 4.91	21.97 ± 4.47	0.003	29.82 ± 4.01	24.96 ± 4.93	<0.001
NAK (ratio)	1.16 ± 0.19	1.18 ± 0.19	0.672	1.16 ± 0.18	1.19 ± 0.23	0.906	1.16 ± 0.27	1.17 ± 0.17	0.922
RF-CSA (cm^2^)	3.51 ± 1.04	2.85 ± 0.65	0.014	3.48 ± 1.02	0.73 ± 0.62	0.019	3.67 ± 0.91	3.27 ± 1.01	0.151
RF-Y-Axis (cm)	1.16 ± 0.29	0.99 ± 0.18	0.012	1.15 ± 0.28	0.98 ± 0.21	0.018	1.20 ± 0.26	1.10 ± 0.28	0.181
Handgrip Strength (HGS, kg)	34.83 ± 9.65	26.53 ± 8.83	0.004	35.40 ± 8.81	19.72 ± 4.75	<0.001	37.00 ± 8.64	31.95 ± 10.04	0.067
TUG (s)	7.63 ± 2.27	8.04 ± 1.77	0.258	7.53 ± 2.10	8.87 ± 2.34	0.048	7.00 ± 1.47	7.86 ± 2.24	0.076
SMI_T12CT (kg/m^2^)	27.67 ± 7.07	22.36 ± 5.01	0.018	27.20 ± 6.93	21.98 ± 5.74	0.061	31.38 ± 9.01	24.79 ± 5.54	0.002
Muscle Area T12 (cm^2^)	79.76 ± 22.64	62.49 ± 12.87	0.015	78.17 ± 22.03	61.53 ± 15.63	0.071	91.48 ± 26.48	70.48 ± 18.18	0.001

Mann–Whitney U test (MWU) used for non-normal distributions (Shapiro–Wilk *p* < 0.05). Student’s *t*-test applied for comparisons with normal distributions (Shapiro–Wilk *p* > 0.05). Values reported as means ± standard deviations for each group, with *p*-values indicating significant differences where applicable. Abbreviations: Phase angle (Pha), standardized phase angle (Spha), fat-free Mass (FFM), body cell mass (BCM), Sodium-to-Potassium Ratio (NAK), rectus femoris cross-sectional area (RF-CSA), rectus femoris Y-axis thickness (RF-Y-Axis), handgrip strength (HGS), Timed Up and Go test (TUG), Skeletal Muscle Index at T12 Level (SMI_T12CT), and muscle area at T12 (cm^2^).

**Table 5:** Morphofunctional muscle mass parameters by obesity and sarcopenic obesity, ESPEN/EASO.

Parameter	Non-Obese (n = 62)	Obese (n = 23)	*p*	Non-Sarcopenic Obese (n = 77)	Sarcopenic Obese (n = 5)	*p*
Pha	4.69 ± 0.70	5.18 ± 0.84	0.008	4.86 ± 0.75	4.24 ± 0.89	0.077
Spha	−1.20 ± 0.97	−0.31 ± 0.49	<0.001	−0.94 ± 0.95	−1.21 ± 1.07	0.546
FFM	53.83 ± 6.75	58.11 ± 8.68	0.019	55.43 ± 7.35	47.92 ± 7.15	0.029
BCM	24.83 ± 4.26	28.69 ± 6.20	0.002	26.21 ± 4.97	20.52 ± 4.98	0.015
NAK	1.22 ± 0.18	1.02 ± 0.12	<0.001	1.17 ± 0.19	1.14 ± 0.19	0.733
RF-CSA	3.27 ± 0.92	3.56 ± 1.19	0.239	3.38 ± 1.01	2.87 ± 0.55	0.300
RF-Y-Axis	1.06 ± 0.25	1.26 ± 0.30	0.004	1.12 ± 0.28	1.06 ± 0.25	0.659
HGS	32.57 ± 9.05	33.78 ± 12.20	0.621	33.95 ± 9.28	16.20 ± 1.79	<0.001
TUG	7.49 ± 2.01	8.23 ± 2.41	0.160	7.53 ± 2.03	10.36 ± 2.26	0.003
SMI_T12CT	24.53 ± 5.60	30.17 ± 8.20	0.003	26.48 ± 6.83	22.11 ± 7.78	0.344
Muscle_area_T12	69.92 ± 17.45	86.98 ± 26.54	0.004	76.06 ± 21.70	59.19 ± 18.52	0.227

Mann–Whitney U test (MWU) used for non-normal distributions (Shapiro–Wilk *p* < 0.05). Student’s *t*-test applied for comparisons with normal distributions (Shapiro–Wilk *p* > 0.05). Values reported as means ± standard deviations for each group, with *p*-values indicating significant differences where applicable. Abbreviations: Phase angle (Pha), standardized phase angle (Spha), fat-free mass (FFM), body cell mass (BCM), Sodium-to-Potassium Ratio (NAK), rectus femoris cross-sectional area (RF-CSA), rectus femoris Y-axis thickness (RF-Y-Axis), handgrip strength (HGS), Timed Up and Go test (TUG), Skeletal Muscle Index at T12 Level (SMI_T12CT), and muscle area at T12 (cm^2^).

**Table 6:** Morphofunctional fat mass parameters by obesity and sarcopenic obesity, ESPEN/EASO.

Parameter	Non-Obese (n = 62)	Obese (n = 23)	*p*	Non-Sarcopenic Obese (n = 77)	Sarcopenic Obese (n = 5)	*p*
FMI	7.29 ± 1.96	11.53 ± 2.46	<0.001	8.18 ± 2.67	12.52 ± 2.13	0.002
L-SAT	0.66 ± 0.38	1.12 ± 0.70	<0.001	0.75 ± 0.48	1.43 ± 0.81	0.031
T-SAT	1.61 ± 0.70	1.85 ± 0.77	0.177	1.63 ± 0.71	2.40 ± 0.59	0.024
S-SAT	0.70 ± 0.29	0.83 ± 0.31	0.078	0.71 ± 0.29	1.03 ± 0.18	0.013
VAT	0.64 ± 0.27	0.67 ± 0.39	0.694	0.64 ± 0.30	0.84 ± 0.36	0.177
TUG	7.49 ± 2.01	8.23 ± 2.41	0.160	7.53 ± 2.03	10.36 ± 2.26	0.006
IMAT_perc_T12	1.79 ± 0.60	2.05 ± 0.88	0.186	1.83 ± 0.68	2.47 ± 0.88	0.166
IMAT_HU_T12	−63.86 ± 5.70	−64.07 ± 4.72	0.889	−63.71 ± 5.34	−66.93 ± 6.08	0.251
VAT_area_T12	167.86 ± 86.98	199.01 ± 63.86	0.176	178.76 ± 83.97	152.73 ± 25.62	0.542
VAT_HU_T12	−96.73 ± 6.66	−99.92 ± 4.77	0.071	−97.71 ± 6.28	−97.13 ± 7.56	0.861
SAT_area_T12	101.15 ± 49.43	162.72 ± 49.34	<0.001	115.97 ± 56.04	166.98 ± 46.43	0.046
SAT_HU_T12	−96.53 ± 9.71	−103.37 ± 7.95	0.011	−98.31 ± 9.95	−101.97 ± 3.60	0.469

Abbreviations: FMI: fat-free mass index; L-SAT: leg subcutaneous adipose tissue; T-SAT: total abdominal adipose tissue; S-SAT: superficial abdominal adipose tissue; VAT: visceral adipose tissue with nutritional echography; TUG: Timed Up and Go test; IMAT_perc_T12: percentage of intramuscular fat tissue at T12 computed tomography level; IMAT_HU_T12: Hounsfield units of intramuscular fat tissue at T12 computed tomography level; VAT_area_T12: area of visceral adipose tissue at T12 computed tomography level; VAT_HU_T12: Hounsfield units of visceral adipose tissue at T12 computed tomography level; SAT_area_T12: area of subcutaneous adipose tissue at T12 computed tomography level; SAT_HU_T12: Hounsfield units of subcutaneous adipose tissue at T12 computed tomography level.

**Table 7:** Univariable analysis of survival and hazard ratios for myoesteatosis at 12 months in patients with IPF.

Parameter	All	HR (Multivariable)	Months	Survival %	95% CI
Absence of myoesteatosis	29 (47.5%)		12	96.6%	100%
Myoesteatosis ≥ 15%	32 (52.5%)	3.13 (1.01–9.70), *p* = 0.049	12	65%	93.8%

Abbreviations: Hazard Ratio (HR), Confidence Interval (CI).

## Data Availability

The original contributions presented in the study are included in the article/Supplementary Materials; further inquiries can be directed to the corresponding authors.

## Supplementary Materials

The following supporting information can be downloaded at: https://www.mdpi.com/article/10.3390/life15040516/s1, Figure S1: Flow chart in idiopathic pulmonary fibrosis.

## Abbreviations

The following abbreviations are used in this manuscript:

ASMM	Appendicular skeletal muscle mass
ASMI	Appendicular skeletal muscle mass index
AUC	Area Under the Curve
BC	Body composition
BCM	Body cell mass
BIVA	Bioelectrical Impedance Vector Analysis
BMI	Body Mass Index
CI	Confidence interval
COPD	Chronic obstructive pulmonary diseases
CT	Computed tomography
DLCO	Diffusing capacity of the lungs for carbon monoxide
DOAJ	Directory of Open Access Journals
ESPEN	European Society for Clinical Nutrition and Metabolism
EASO	European Association for the Study of Obesity
FFM	Fat-free mass
FMI	Fat-free mass index
GLIM	Global Leadership Initiative on Malnutrition
HGS	Handgrip strength
HR	Hazard ratio
HU	Hounsfield unit
IPF	Idiopathic pulmonary fibrosis
IMAT	Intramuscular adipose tissue
LD	Linear dichroism
L-SAT	Leg subcutaneous adipose tissue
MDPI	Multidisciplinary Digital Publishing Institute
MFA	Morphofunctional assessment
NU	Nutritional ultrasound^®^
CRP	C-reactive protein
Pha	Phase angle
SPha	Standardized phase angle
RF-CSA	Rectus femoris cross-sectional area
RF-Y-axis	Rectus femoris Y-axis
ROC	Receiver Operating Characteristic
SAT_area_T12	Area of subcutaneous adipose tissue at the T12 computed tomography level
SAT_HU_T12	Hounsfield units of subcutaneous adipose tissue at the T12 computed tomography level
SMI_T12CT	Skeletal Muscle Area in computed tomography at the T12 level
S_SAT	Superficial abdominal adipose tissue
TBW	Total Body Water
T12-CT	Computed tomography at the T12 level
TLA	Three letter acronym
T-SAT	Total abdominal adipose tissue
TUG	Timed Up and Go test
VAT	Visceral adipose tissue
VAT_area_T12	Area of visceral adipose tissue at the T12 computed tomography level
VAT_HU_T12	Hounsfield units of visceral adipose tissue at the T12 computed tomography level
