# Supplementary figures and images for: The Nutritional Phenotyping of Idiopathic Pulmonary Fibrosis Through Morphofunctional Assessment: A Bicentric Cross-Sectional Case–Control Study
Source: Life (Basel). 2025 Mar 21;15(4):516. doi: 10.3390/life15040516 (PMC12029122; doi:10.3390/life15040516)

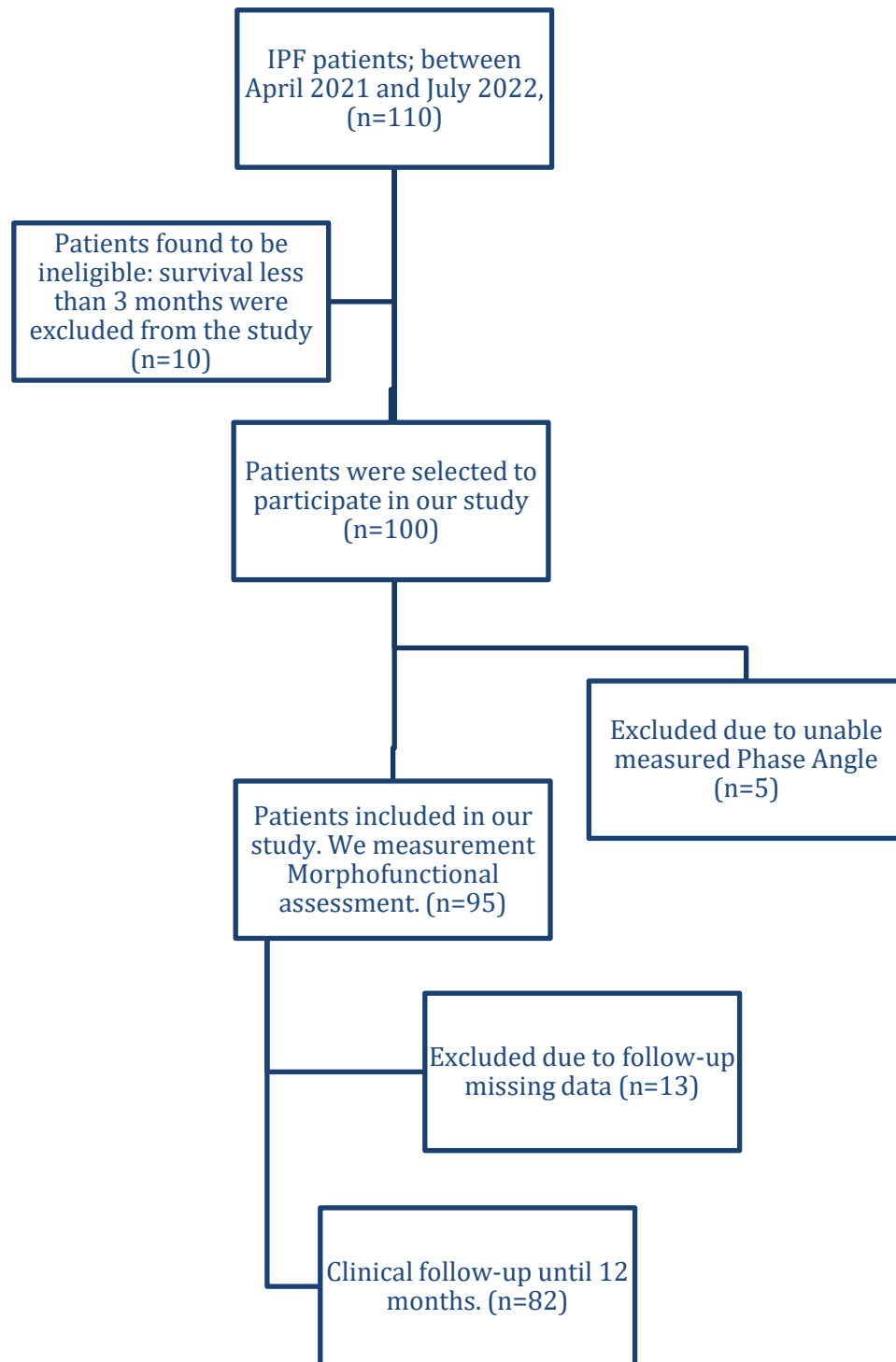

Abbreviations: IPF: idiopathic pulmonary fibrosis.

Supplement: Supplementary file 1 [file life-15-00516-s001.zip › life-3486362-supplementary.pdf]
